# An Unsupervised Brain Extraction Quality Control Approach for Efficient Neuro-Oncology Studies

**DOI:** 10.1007/s10278-025-01570-y

**Published:** 2025-06-25

**Authors:** Sarthak Pati, Stefan Wagner, Siddhesh Thakur, Evan Calabrese, Russell Shinohara, Spyridon Bakas

**Affiliations:** 1 Division of Computational Pathology, Department of Pathology and Laboratory Medicine, Indiana University School of Medicine, Indianapolis, IN USA; 2 Center for Federated Learning in Precision Medicine, School of Medicine, Indiana University, Indianapolis, IN USA; 3 Medical Working Group, MLCommons, San Francisco, CA USA; 4 Drexel University, Philadelphia, PA USA; 5 Duke University, Durham, NC USA; 6 Penn Statistics in Imaging and Visualization Center, Department of Biostatistics, Epidemiology, and Informatics, University of Pennsylvania, Philadelphia, USA

**Keywords:** Automated quality control, Brain mask, Neuro-oncology, Clustering, AI

## Abstract

Brain extraction is essential in neuroimaging studies for patient privacy and optimizing computational analyses. Manual creation of 3D brain masks is labor-intensive, prompting the development of automatic computational methods. Robust quality control (QC) is hence necessary for the effective use of these methods in large-scale studies. However, previous automated QC methods have been limited in flexibility regarding algorithmic architecture and data adaptability. We introduce a novel approach inspired by a statistical outlier detection paradigm to efficiently identify potentially erroneous data. Our QC method is unsupervised, resource-efficient, and requires minimal parameter tuning. We quantitatively evaluated its performance using morphological features of brain masks generated from three automated brain extraction tools across multi-institutional pre- and post-operative brain glioblastoma MRI scans. We achieved an accuracy of 0*.*9 for pre- and 0*.*87 for post-operative scans, thus demonstrating the effectiveness of our proposed QC tool for brain extraction. Additionally, the method shows potential for other tasks where a user-defined feature space can be defined. Our novel QC approach offers significant improvements in flexibility and efficiency over previous methods. It is a valuable tool, targeting reassurance of brain masks in neuroimaging and can be adapted for other applications requiring robust QC mechanisms.

**Supplementary Information:**

The online version contains supplementary material available at 10.1007/s10278-025-01570-y.

## Introduction

Neuroimaging and neuro-oncology AI studies are pivotal in advancing our understanding of brain pathologies and developing effective treatments. A critical step in these studies is the extraction of brain images, which serves two primary purposes: deidentification and optimization of downstream AI methods. Deidentification ensures patient privacy by removing identifiable information from the images, thereby complying with ethical standards and legal regulations. Additionally, brain extraction enhances the performance of AI algorithms by focusing on relevant anatomical structures, thus improving the accuracy and efficiency of diagnostic and prognostic models. These processes are essential for leveraging the full potential of AI in neuroimaging and neuro-oncology research [1–3].

Brain masks are binary, volumetric images crucial for discriminating the brain tissue from non-brain elements, serving as a key preprocessing step to enhance model performance and ensure patient privacy compliance [4]. The labor-intensive nature of manually creating these masks [5] has driven significant research into computational methods for automating brain masking [4, 6–14]. Despite such advancements, inherent imperfections in these algorithms necessitate a robust quality control (QC) system [15].

QC is vital in both clinical studies and computational research, as data quality significantly impacts the development and evaluation of AI methodologies [16, 17], which are increasingly prevalent and high-performing in brain extraction [4, 14]. Traditional metrics like the Dice Similarity Coefficient, Hausdorff Distance, and Jaccard Index are commonly used to evaluate brain extraction performance [18]. However, these metrics may not capture all necessary information, especially in complex tasks.

Domain-specific AI quality assessment tools, such as PathProfiler [19], offer potential but are limited by the need for large training datasets and domain-specific retraining. Previous approaches in segmentation evaluation [20–22] have explored various methodologies, including uncertainty metrics and synthesis-based evaluations. However, these methods often remain confined within specific domains, limiting their flexibility and applicability.

Recent advancements in MRI quality control (QC) have leveraged machine learning techniques to enhance the accuracy and efficiency of image assessment. The UK Biobank study [23] utilized supervised learning with a machine learning classifier specifically optimized for their T1-weighted dataset, demonstrating the potential of tailored QC approaches. Similarly, Rosen et al. [24]. employed cortical surface reconstruction from FreeSurfer as a basis for QC, although this method proved computationally expensive. They highlighted the variability of FreeSurfer metrics depending on the scanner used, necessitating ground truth annotations for effective tuning. Monereo et al. [25]. compared various QC strategies for brain segmentation, noting that methods relying on morphological features extracted with FreeSurfer and outlier detection using Tukey’s method performed poorly, partly due to the simplistic modeling of features as single Gaussians. In contrast, the Qoala-T study [26] tested different classifiers on FreeSurfer morphometric features, finding that random forest classifiers yielded the best performance in supervised QC tasks. Our proposed method allows for a flexible QC approach that can be tuned on a use-case basis without requiring any training. Other methods like MRIQC [27] and MRQy [28] are tailored for MRI and fMRI images and are not well-suited for segmentation QC. While MRIQC uses pre-trained random forest classifiers to mitigate training hurdles, it still requires labeled and diverse training data. While MRQy uses unsupervised clustering similar to our method, earlier machine learning-based approaches to out-of-distribution detection [29] require specific model training.

To address these challenges, we propose an adaptable and flexible QC approach. Our method integrates custom feature selection with minimal parameter tuning, designed for domain-optimized performance with a modest computational footprint. For the specific use case (i.e., QC of brain masks), we have been inspired by statistical outlier detection paradigms [29–31], and we demonstrate our approach relying solely on segmentation labels, eliminating the need for raw imaging data. This universally applicable QC method can be customized for various domain-specific applications based on the selected features, whether they come from a segmentation label or from multiple imaging modalities.

## Methods

### Data

For our study and evaluation of our methodology, we rely on 966 cases, each of them described by both an expertly curated ground truth brain mask and a computationally generated mask. Our set of ground truth brain masks, as depicted in Table 1, came from multiple institutions, namely, the University of Pennsylvania Health System (453 cases), Thomas Jefferson University (152 cases), MD Anderson Cancer Center (29 cases), The Cancer Genome Atlas—TCGA [32] (132 cases), and Duke University (200 cases), which in aggregate cover both pre-operative (Pre-Op) as well as post-operative (Post-Op) brain mask segmentations. The data from Duke was used as a completely independent out-of-sample analysis of our method. Each subject needed to have all the 4 structural modalities (i.e., native T1, post-contrast T1-weighted (T1Gd), native T2-weighted (T2), and T2-weighted Fluid Attenuated Inversion Recovery (FLAIR)) and each scan needed to be 3D (either native or slices) to be included in the study. The processing of the data followed the protocol followed in the Brain Tumor Segmentation challenge [33].

**Table 1:** Number of ground truth pre-operative (Pre-Op) and post-operative (Post-Op) masks available from different sites

Site	Pre-Op	Post-Op	Total
University of Pennsylvania	453	-	453
Thomas Jefferson University	152	-	152
MD Anderson Cancer Center	29	-	29
The Cancer Genome Atlas—TCGA	132	-	132
Duke University	100	100	200
Total	866	100	966

For computational generation of brain masks, we utilized three distinctly different forms of skull-stripping algorithms, namely the Brain Extraction Tool (BET) [13], HD-BET [14], and BrainMaGe [4]. Cumulatively, our set contains 966 ground truth masks, 966 BET-generated masks, 7728 masks generated via BrainMaGe and HD-BET, and 5796 fusions. An example of these methods along with their associated ground truths can be seen in Fig. 3. We employed HD-BET and BrainMaGe to generate brain masks for subjects across various structural MRI modalities (T1, T1Gd T2, and FLAIR). This resulted in the construction of four masks per case for HD-BET and BrainMaGe each, one for each modality. We generated one additional brain mask for all subjects in our dataset using the native T1 modality for BET, since this method was designed to only work with T1 [13]. Lastly, we also utilized three different label fusion approaches (further detailed in the “Label Fusion” section), granting us an additional six generated masks per subject through HD-BET and BrainMaGe modality fusions.

### Label Fusion

Label fusion is the process of combining multiple segmentations of the same subject into a collective or consensus result. This has shown to provide a better overall annotation compared to using the result from a single method [34, 35]. We utilized majority voting [36], simultaneous truth and performance level evaluation (STAPLE) [37], and selective and iterative method for performance level estimation (SIMPLE) [35]. Each fusion segmentation was created using the segmentations of each of the imaging modalities, i.e., T1, T2, T1Gd, and FLAIR [38].

### Our Method

Our method provides a robust approach to brain mask quality evaluation to perform automated QC without requiring ground truth annotations. We use the statistical feature distribution of a chosen ground truth set *B* to serve as a baseline for the QC method. By leveraging morphological feature extraction, *k*-means clustering, and Mahalanobis distance calculations, we can assess the similarity of any data point to our baseline set *B* and flag masks with high fault potential. The following sections delve into the different facets of our algorithm, beginning with the pivotal stage of feature extraction.

### Feature Extraction

The unsupervised nature of our algorithm underlines the importance of feature extraction in prediction accuracy. For our brain mask implementation, we utilized SimpleITK [39] to extract [23] morphological features [40–45] that encapsulate the key spatial and structural characteristics of brain masks.

In order to accurately and efficiently approximate all features even in higher dimensions, SimpleITK makes use of the gamma function for evaluating factorials of positive integers [46], achievable through its definition

1 $$\Gamma \left(z+1\right)={\int }_{0}^{\infty }{t}^{z}{e}^{-t}dt=z!$$

Other more complex features, such as ellipsoid diameter, are defined via central moments *I* and their eigenvalues [47], which in our 3D discrete space can be summarized as

2 $$Ia{a}{\prime}=-\frac{1}{m}\left\{\sum_{i=1}^{N}{m}_{i}\left[\left({\overline{x} }_{i}-{\overline{x} }_{0}\right)\bullet \widehat{n}\right]\bullet \left[\left({\overline{x} }_{i}-{\overline{x} }_{0}\right)\bullet \widehat{n}{\prime}\right]\right\}$$

where *N* is the number of pixels within the shape, *x*¯_0_ is a vector describing the geometric reference point, *x*¯_*i*_ and *m*_*i*_ are the location and weight of pixel *i*, respectively, and *m* is the sum of all weights. *n*ˆ and *n*ˆ^′^ are the normal unit vectors with respect to our specified Cartesian planes *α* and *α*^′^, i.e., the x-, y-, or z-axis, which allow us capture all central moments for our (*x, y, z*) Cartesian system *G* in the following symmetric matrix.

3 $${\sigma }_{G}=\left[\begin{array}{ccc}{I}_{11}& {I}_{12}& {I}_{13}\\ {I}_{21}& {I}_{22}& {I}_{23}\\ {I}_{31}& {I}_{32}& {I}_{33}\end{array}\right]$$

with three eigenvalues *λ*_1_, *λ*_2_, and *λ*_3_, as well as three eigenvectors *e*_*λ,*1_, *e*_*λ,*2_, and *e*_*λ,*3_. A full definition of all used features can be found in Supplementary Table 5.

After computation, all features are normalized to ensure comparability during the clustering phase by removing their mean and scaling to unit variance.

### Clustering

With normalized features, we perform clustering on the ground truth set *B*, with the resulting clusters serving as reference points for later similarity calculations. We apply *k*-means for this task and determine the optimal number of clusters *k* by executing 500 iterations of clustering for each possible value of *k*, evaluating the best clustering with average Rand Index scores [48]. We have observed that for our use case, the ideal clustering determined via Rand Index scoring aligns with the desired sensitivity metrics of our QC implementation. For further details, we ask the reader to refer to Fig. 6. The range of *k* values considered spans from 2 to 10, inclusive. We then use the Mahalanobis distance for evaluating similarity.

### Mahalanobis Distance

In contrast to Euclidean distance, Mahalanobis distance [49] incorporates standard deviation when evaluating the distance between a data point and another complete data cohort. This parametric metric determines the distance of a point from a distribution by taking into account the mean and the covariance matrix. It estimates probability density using measurements from Gaussian-distributed data, which is a reasonable assumption for the extensive, multi-dimensional feature spaces examined in this context. Within this context, it can be defined as follows:

4 $$d\left(x,Y\right)=\sqrt{{\left(x-\overline{Y }\right)}^{T}{S}_{Y}^{-1}\left(x-\overline{Y }\right)}$$

where *x* is a vector of *n* dimensions, *Y* is a set of vectors with *n* dimensions, and *S*_*Y*_ is the covariance matrix of *Y*. Given its definition and properties, it is often used for statistical outlier detection [50].

Similar to prior work in this space [29], we apply the Mahalanobis distance to measure the similarity of a given point to our ground truth sets from a data point *v* to each of the *k* ground truth clusters, *C*_*i*_. This provides an indication of how close a given data point is to the existing feature distributions of the ground truth data. Subsequently, we define and calculate the weighted mean and minimum Mahalanobis distances for each vector *v* for future computations as:

5 $${d}_{\mathrm{min}}\left(v\right)=\underset{i}{\mathrm{min}}d\left(v,{C}_{i}\right)$$

6 $$\overline{d }\left(v\right)=\sum_{i=1}^{k}\frac{d\left(v,{C}_{i}\right)}{\left|{C}_{i}\right|}$$

where *d*_min_(*v*) and *d*^¯^(*v*) represent the minimum and weighted mean Mahalanobis distance of vector *v*, respectively.

### Similarity Metric r

In order to create our method of similarity evaluation, we use the abovementioned metrics to form two baseline sets *D*_min_ and *D*_*µ*_.

7 $${D}_{\mathrm{min}}=\left\{{d}_{\mathrm{min}}\left(b\right):b\in B\right\}$$

8 $${D}_{\upmu }=\left\{\overline{d }\left(b\right):b\in B\right\}$$

We then derive threshold values *t*_min_ and *t*_*µ*_ by selecting the *n*th percentile of both *D*_min_ and *D*_*µ*_ in terms of minimum values. This allows us to define our similarity metric as

9 $$r=\frac{\pi }{2}\mathrm{arctan}\left(\gamma *\frac{{d}_{\mathrm{min}}\left(v\right)}{{t}_{\mathrm{min}}}+\left(1-\gamma \right)*\frac{\overline{d }\left(v\right)}{{t}_{\mu }}\right)$$

where *γ* is a weighing parameter and *v* is the vector for which we want to establish its similarity *r* to the ground truth set *B*.

The only remaining aspect for QC is establishing a cutoff value *r*_max_, such that any subject *s* is considered to pass only if *r*^*s*^ ≤ *r*_max_. By virtue of its definition, an *r*_max_ of 0.5 is sensible with *r* < 0*.*5 and *r* > 0*.*5 typically indicating strong and weak similarity to the ground truth, respectively. For stricter cutoffs, it is advisable to lower the percentile regions for determining *t*_min_ and *t*_*µ*_ as they directly shift the distribution of the *r* value. For our use case, we use *r*_max_ = 0*.*5, with the 90 th percentile of both *D*_min_ and *D*_*µ*_ to set *t*_min_ and *t*_*µ*_.

### Alternatives and Variations

Our method offers flexibility in tailoring cutoffs and in scaling the combined metrics of the minimum and weighted mean via the *γ* factor. We theorize that local cluster similarity, as provided by the minimum Mahalanobis distance, holds equal if not greater importance than global similarity conferred by the weighted mean. For instance, if there exists an outlier cluster of several ground truth images, data points highly similar to this cluster should not be dismissed outright for not conforming to the average trend of the data. However, these data points, similar to outlier clusters, should still be scrutinized more rigorously than those samples that are equally similar to “main-stream” clusters. For this reason, we argue that *γ* > 0*.*5 without completely neglecting the importance of global similarity is a reasonable choice for our problem domain. However, applying our approach to a different problem set might not allow these assumptions to always hold. Depending on the nature of the data, as well as the desired FN and FP rates, experimentation with varying values of *γ* alongside different percentile cutoffs will most likely be required for ideal QC behavior. In our experiments, we decided to use *γ* = 0*.*75.

While our methodology mainly revolved around the minimum and weighted mean Mahalanobis distances, other statistical measures could also be employed. For instance, the median Mahalanobis distance could be used as a replacement for the weighted mean Mahalanobis distance. One may even use other statistical metrics, or any arbitrary combination of them. As long as the weighted sum of the metrics when calculating *r* reflects the desired balance of both local and global similarity, the essence of the algorithm would not be compromised.

### Experimental Design

#### Evaluation Metrics

As part of our experiment, we utilize two common segmentation metrics in order to aid in our performance evaluations, “Dice Similarity Coefficient” (henceforth referred to as simply “Dice”) [51] as well as a 95 th percentile “Hausdorff Distance” [52].

While the “Dice” score represents a global measure of overlap, “Hausdorff Distance” quantifies the distance between the boundaries of the ground truth labels against the predicted labels, making it very sensitive to local differences. As such both metrics complement each other well for evaluating segmentation performance, which proves crucial for establishing a ground truth set of guaranteed segmentation failures.

This set of guaranteed segmentation failures is what we consider our sanity set. By containing segmentations that are expected to be flagged during evaluation, the sanity set is constructed as a tool to help us evaluate the performance of our QC method. This set consists of all masks generated using BET, HD-BET, and BrainMaGe, alongside their fusions, with a “Dice” score below 0*.*95 or that exceed a 95 th percentile “Hausdorff Distance” of 15 when compared to their corresponding ground truth segmentations. This allows us to categorize our models’ predictions into true positives (TP), false positives (FP), true negatives (TN), and false negatives (FN), with negatives and positives expected to pass and fail QC, respectively. Of the 1861 generated brain masks within the sanity set, 354 were from BET, 359 from HD-BET and its fusions, and 1148 from BrainMaGe and its fusions.

After acquiring these labels through evaluation via our sanity set, we rely on the traditional machine learning classification metrics sensitivity, specificity, precision, and accuracy [53] for a detailed performance analysis of our QC method to classify between acceptable and unacceptable brain masks.

Sensitivity, often termed recall, quantifies the proportion of true positive subjects correctly identified by our model. Given the medical domain’s imperative to minimize false negatives, this metric is indispensable for assessing the effectiveness of our methodology.

Specificity, conversely, measures the ability of our method to correctly identify true negative subjects. While in clinical QC scenarios, the accurate classification of negatives may be deemed of lesser importance compared to positives, specificity remains a pertinent indicator of our model’s comprehensive performance.

Precision captures the proportion of subjects identified as positive that are indeed true positives. It complements sensitivity: while it is theoretically possible to achieve perfect sensitivity at the expense of precision, a diminished precision score detracts from the overall utility of our QC method.

Lastly, we consider accuracy, which denotes the overall rate of correct classifications across all subjects. As such, it serves as a universally applicable metric for evaluating classification models.

#### Experiments

We used the ground truth annotations as our baseline set *B* to calculate Mahalanobis distances and our similarity metric *r*, as outlined in the previous sections (the “Similarity Metric r” section) by relying on 23 morphological features (the “Feature Extraction” section). For our algorithm parameters, we used the 90 th-percentile for setting *t*_min_ and *t*_*µ*_, used *γ* = 0*.*75, and let *r*_max_ = 0*.*5. With each generated mask *v* having been assigned its own *r* value, we classify a mask as faulty if *r* > *r*_max_. The sanity set is then utilized for performance metrics, using it as a ground truth for classifying faulty masks within our dataset. We measure the effectiveness of our method through sensitivity, specificity, accuracy, and precision.

To further highlight the statistical properties of our methodology, we conducted an experiment exclusively on ground truth segmentations. We applied our QC approach to evaluate the similarity between pre- and post-operative ground truth masks contained in the Duke University dataset in two distinct runs. We maintained the same parameters as in our preceding experiment.

## Results

**Fig. 1:**
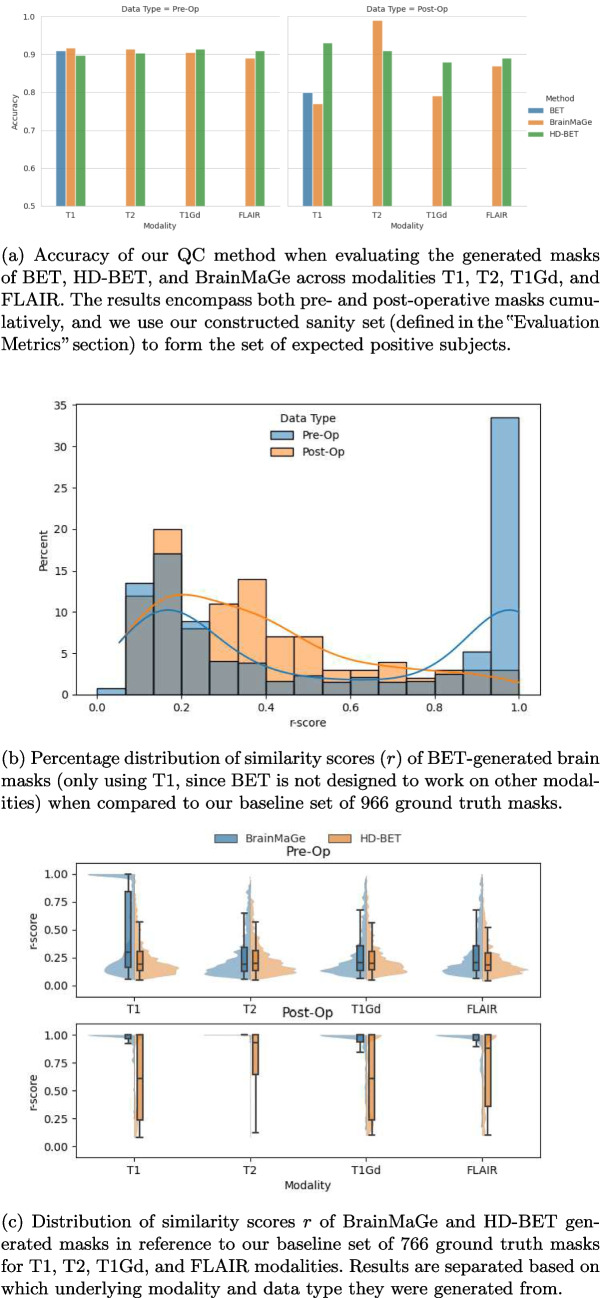
Results of our QC method when evaluating the generated masks of BET, HD-BET, and BrainMage

In order to assert the validity of our approach, we first conducted a comprehensive assessment of the accuracy of our QC method across different mask generation methods and modalities. We evaluate BET performance on only T1, and both HD-BET [14] and BrainMaGe [4] on T1, T2, T1Gd, and FLAIR. We evaluate using our aforementioned sanity set (defined in the “Evaluation Metrics” section) to determine ground truth labels and use the similarity score *r* to classify via an *r*_max_ ≈ 0*.*5 cutoff. We distinguish between pre- and post-operative performance since the shape characteristics of the post-operative brains could potentially be unique, which is important to capture. The results are illustrated in Fig. 1a. An equivalent visualization for fusion performance may be found in Fig. 4 in the supplementary.

For further clarity, we use our remaining evaluation metrics (defined in the “Evaluation Metrics” section) to conduct a cumulative assessment and comparison between pre- and post-operative performance across all methods, modalities, and fusions. We additionally provide the metrics across the total of all generated masks. The results of classifying all of our 12,990 and 1500 generated segmentations for pre- and post-operative cases, respectively, based off the sanity set labels can be found in Table 2.

**Table 2:** Cumulative performance of our methodology for BET, HD-BET, and BrainMaGe across T1, T2, T1Gd, and FLAIR modalities as well as their fusions. Performance evaluation was separated by data type, and we utilized our sanity set (defined in the “Evaluation Metrics” section) to form the set of expected positive subjects

Metric	Pre-Op	Post-Op	Total
Accuracy	0.9083	0.8753	0.9048
Sensitivity	0.9504	0.9978	0.9736
Specificity	0.9049	0.6853	0.8947
Precision	0.4408	0.8310	0.5768

Additionally, we leveraged the predictive results from our QC method to analyze the scoring of generated masks among BET, HD-BET, and BrainMaGe when compared against their ground truth counterparts. With low and high *r* values indicating strong and weak statistical correlation respectively, we are able to define how well BET, HD-BET, and BrainMaGe perform across modalities according to our approach. The *r-score* distribution across the pre- and post-operative masks generated through BET is depicted in Fig. 1b. Results for BrainMaGe and HD-BET across T1, T2, T1Gd, and FLAIR modalities can be seen in Fig. 1c. Equivalent fusion results can be found in Fig. 5 in the supplementary.

For an encompassing analysis, we also evaluated the practical performance of each brain mask generation method via our QC methodology. We showcase the results from our cutoff-based binary classification method by categorizing each mask with a score of *r* > *r*_max_ = 0*.*5 as faulty a segmentation. Our findings are summarized in Table 3 for each modality, as well as in Table 4 in the supplementary for our three fusion approaches.

**Table 3:** Pass rate of pre- and post-operative generated masks for each modality-method pair when using our proposed QC method (defined in the “Our Method” section). Results are an accumulation of Fig. 1b and c, where each generated mask has their similarity score compared to *r*_max_ = 0*.*5. Generated masks with a similarity score *r* ≤ *r*_max_ are considered to have passed QC

		**BET**	**HD-BET**	**BrainMaGe**
Pre-Op	T1	0.5103	0.8949	0.6293
	T2	-	0.9064	0.8568
	T1Gd	-	0.9133	0.8464
	FLAIR	-	0.9133	0.8418
Post-Op	T1	0.7400	0.4900	0.0200
	T2	-	0.2300	0.0000
	T1Gd	-	0.4500	0.0800
	FLAIR	-	0.3700	0.0500

It is also important to underscore that our proposed QC methodology is not inherently constrained to only differentiating between high and low-quality brain masks. Its design principles are rooted in maintaining a degree of feature consistency in alignment with ground truth segmentations, which consequently imparts it with QC characteristics.

**Fig. 2:**
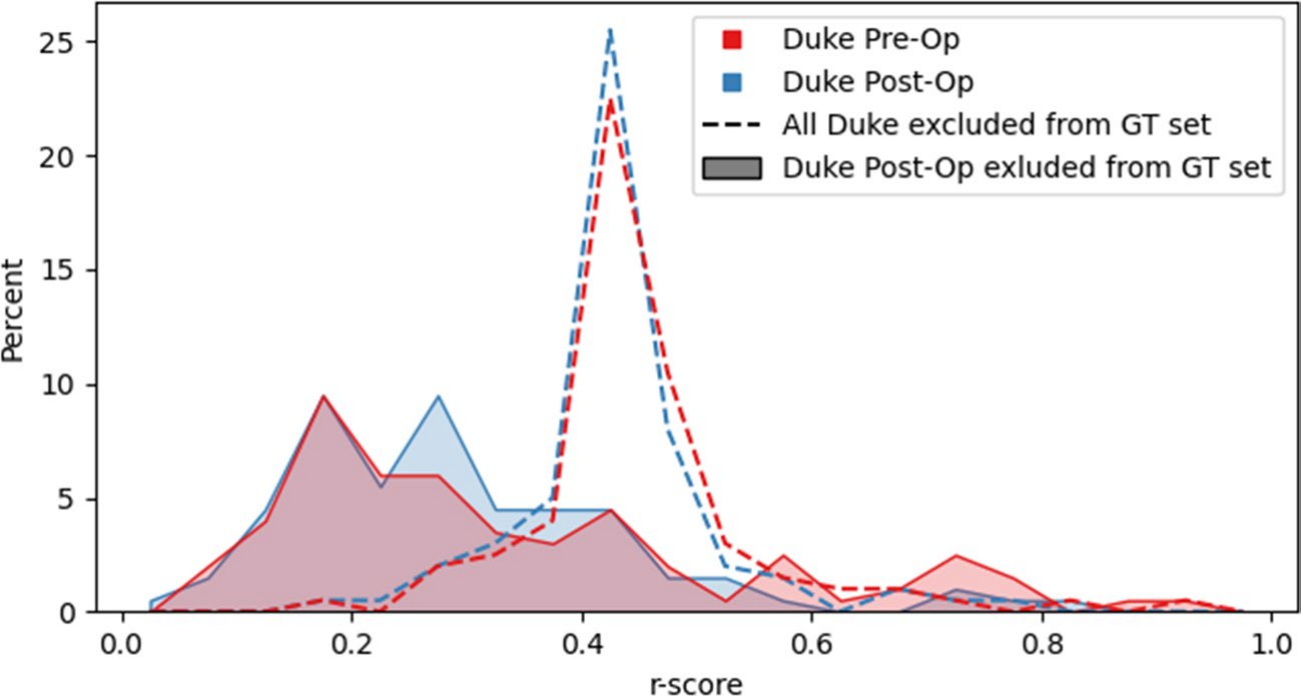
Percentage distribution of similarity scores *r* of pre- and post-operative ground truth (GT) segmentations from the Duke University dataset. Similarity was assessed (1) against a baseline set containing all ground truth masks from the remaining institutions (*n* = 766), and (2) a baseline set containing all pre-operative ground truth masks (*n* = 866), i.e., all ground truth masks excluding post-operative masks from Duke University

To further visualize this, we experimented with applying our QC method on our multi-institutional ground truth segmentations. In particular, we highlight the dynamics of the Duke University data cohort, as depicted in Fig. 2. We compare the similarity scores of the pre- and post-operative ground truth segmentations from Duke University for two cases: initially excluding all Duke University dataset ground truth masks from the baseline set and subsequently incorporating half of them, constructing a fully pre-operative baseline set.

## Discussion

In this manuscript, we present a novel automated brain mask QC approach that integrates feature extraction with clustering and statistical distance metrics to estimate quality based on ground truth annotations. Our method requires no training and relies solely on morphological features derived from the brain masks, eliminating the need for actual brain imaging data. Our curated sanity set demonstrates the method’s efficacy, showcasing its speed and flexibility, and paving the way for future advancements in QC.

**Fig. 3:**
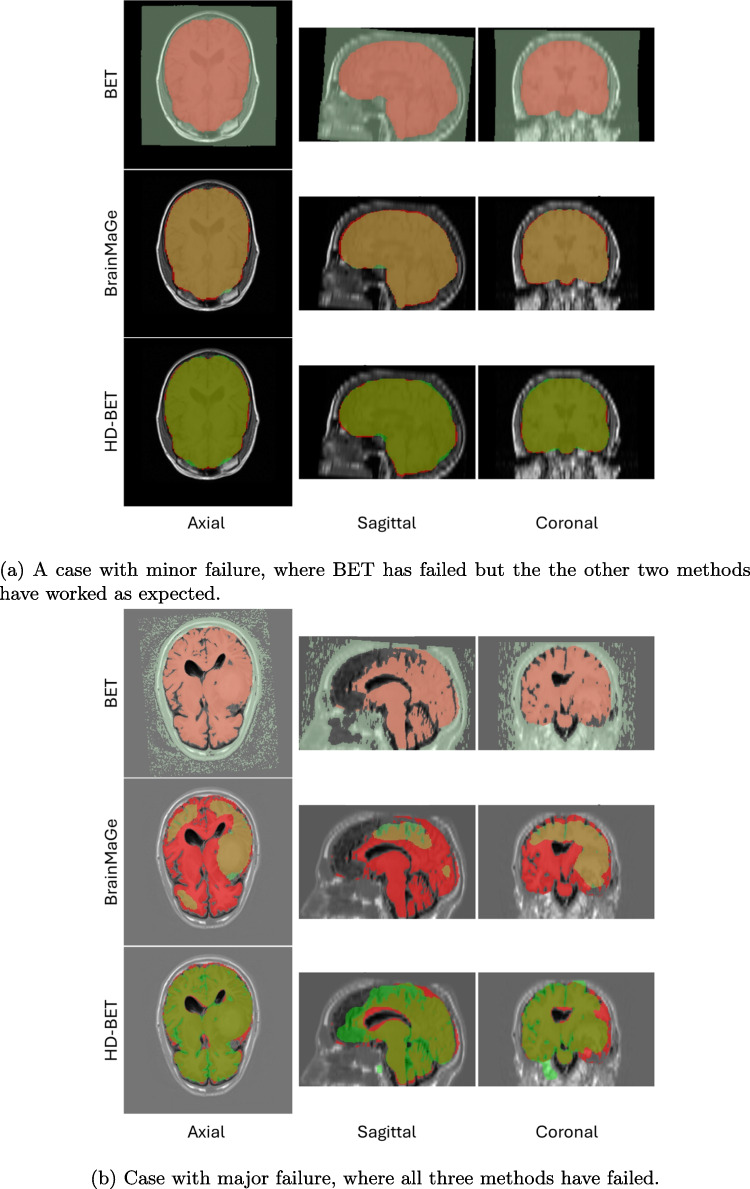
Screenshots representing the extent of quality control issues with brain extraction methods. The red overlay represents the ground truth, and each of the separate colors represents a method (BET, BrainMage, and HD-BET, respectively)

Existing QC methodologies such as MRIQC [27] and MRQy [28] are specifically designed for MRI and fMRI images, but they fall short when applied to QC of segmentations or annotations. These methods either depend on supervised learning [23], necessitating annotations and training, or employ overly simplistic outlier detection techniques [25] that often yield suboptimal results. In contrast to prior work [23–28], our proposed method distinguishes itself by utilizing multivariate morphological descriptors and extending outlier detection beyond single Gaussian distributions for the segmentation of brain masks. Notably, our approach requires no training, allowing for the effortless expansion of the ground truth dataset used as a basis for QC in real-world environments. Furthermore, the versatility of our method enables its extension to multiple applications, provided the feature space is appropriately defined, even though here we showcase its performance for the application of brain extraction. This innovative approach addresses the limitations of existing QC methodologies and offers a robust, adaptable, and generalizable solution for QC for various applications. Figure 1b, c, and Table 3 reveal that masks generated by both HD-BET and BrainMaGe for pre-operative instances align closely with ground truth segmentations (with an advantage to HD-BET because of consistency). For post-operative scenarios comparing only T1 scans, BET performs the best. The performance differences between HD-BET and BrainMaGe in pre-operative subjects, especially for T2, T1Gd, and FLAIR modalities, highlight the sensitivity of our method in checking the quality of the mask, which quantifies segmentation failures based on morphological features (Fig. 3).

We also evaluated our method on a completely out-of-sample dataset from Duke University (see the “Data” section for details). As illustrated in Fig. 2, it shows consistent score distributions across pre- and post-operative data in this dataset as well, suggesting that statistical attributes of the datasets influence our feature space more than the distinction between pre- and post-operative masks. This indicates that our method can maintain data consistency, reducing statistical anomalies and improving algorithmic accuracy.

While our proposed generalizable QC method offers significant advantages in terms of flexibility and adaptability, it is not without limitations. One inherent drawback is that it can never achieve 100% accuracy, as outlier detection relies on flagging a certain percentile of ground truth features, which may inadvertently include valid data points. Additionally, the performance of our method is likely to be inferior to that of supervised approaches, which benefit from training on annotated datasets. This tradeoff between flexibility and performance necessitates further experimentation to fully understand its extent and implications. Finally, the proposed method is inherently dependent on the feature space that was used to describe the use-case or application at hand, and hence the feature space should be representative of the use-case. For example, if the application is related to assessing annotations and the user passes first order intensity or second order radiomic features, then the feature space will not be representative of the task at hand, and thus the method will not produce appropriate results. Despite these limitations, the method’s ability to operate without labels or training and its potential for extension to various applications make it a valuable tool in the realm of QC.

We showcase our approach’s adaptability in feature extraction as a strength, it can also highlight unnecessary statistical variations. For example, mask smoothness and pixelation, which are less critical for clinical evaluations, can affect score trends. Also, the chosen features can dramatically impact the algorithm’s performance and may induce bias in prediction trends by unintentionally creating feature-outcome correlations. Despite these limitations, our method offers a comprehensive review of data cohesion and has potential for various applications beyond QC, such as outlier detection and out-of-distribution testing between two datasets, where a quantitative assessment of feature sets from two independent datasets are needed, and can be critical for validating the generalizability and robustness of computational methods [54].

## Conclusion

In conclusion, our method represents a significant advancement in brain segmentation quality control. By integrating feature extraction with statistical methods, we provide a flexible approach that aids in quality estimation and data consistency. Future work should refine feature selection for different domains and test the approach across varied datasets, underscoring its potential for broad application.

## Supplementary Information

Below is the link to the electronic supplementary material.

Supplementary file1 (DOCX 202 KB)

## Data Availability

Information related to the data and code used for this manuscript is available at https://github.com/IUCompPath/QC_MRI.
