# Supplementary material for: An Unsupervised Brain Extraction Quality Control Approach for Efficient Neuro-Oncology Studies
Source: J Imaging Inform Med. 2025 Jun 25;39(2):1608–18. doi: 10.1007/s10278-025-01570-y (PMC13103071; doi:10.1007/s10278-025-01570-y)
Supplement: Supplementary file 1 — Supplementary file1 (DOCX 202 KB) [file 10278_2025_1570_MOESM1_ESM.docx]

# Supplementary

**
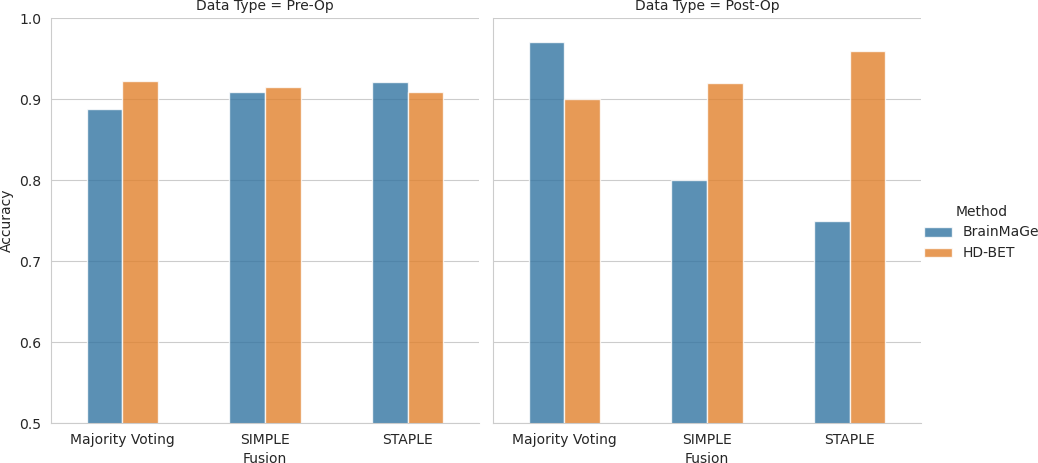
**

Fig. 4: Accuracy of our QC method when evaluating the generated masks of BET, HD-BET, and BrainMaGe across Majority Voting, SIMPLE, and STAPLE fusions. The results encompass both pre- and post-operative masks cumulatively, and we use our constructed sanity set (defined in Section 4.4.1) to form the set of expected positive subjects.


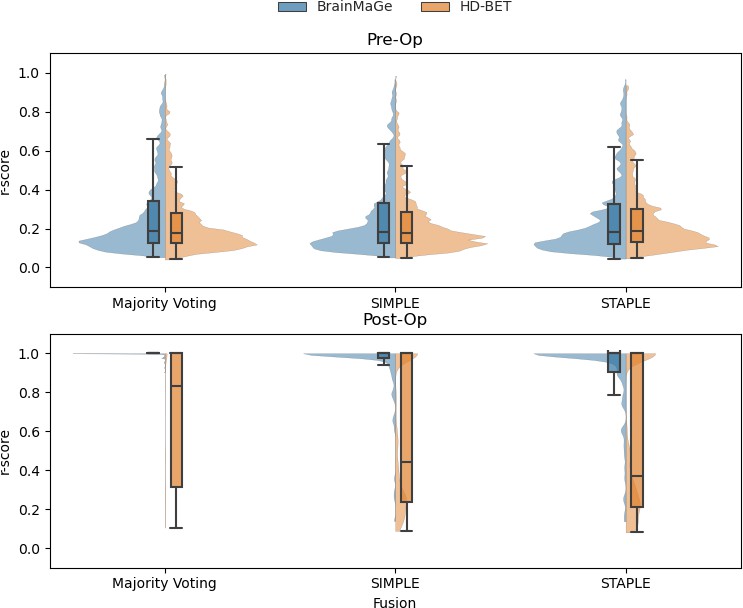


Fig. 5: Distribution of similarity scores *r* of BrainMaGe and HD-BET generated masks in reference to our baseline set of 966 ground truth masks for Majority Voting, SIMPLE, and STAPLE fusions. Results are separated based on which underlying fusion and data type they were generated from.

Table 4: Pass-rate (defined as simply *N_passed_/N_total_* where *N* is the number of data points) of pre- and post-operative generated masks for each fusion-method pair when using our proposed QC method (defined in Section 4.3). The results utilized are equivalent to Figure 5, where each generated mask has their simi- larity score compared to *r_max_* = 0*.*5. Generated masks with a similarity score *r <*= *r_max_* are considered to have passed QC.

|  | | **HD-BET** | **BrainMaGe** |
| --- | --- | --- | --- |
| **Pre-Op** | Majority Voting | 0.9237 | 0.8568 |
|  | SIMPLE | 0.9157 | 0.8579 |
|  | STAPLE | 0.9099 | 0.8602 |
| **Post-Op** | Majority Voting | 0.4200 | 0.0000 |
|  | SIMPLE | 0.5200 | 0.0400 |
|  | STAPLE | 0.5700 | 0.0700 |


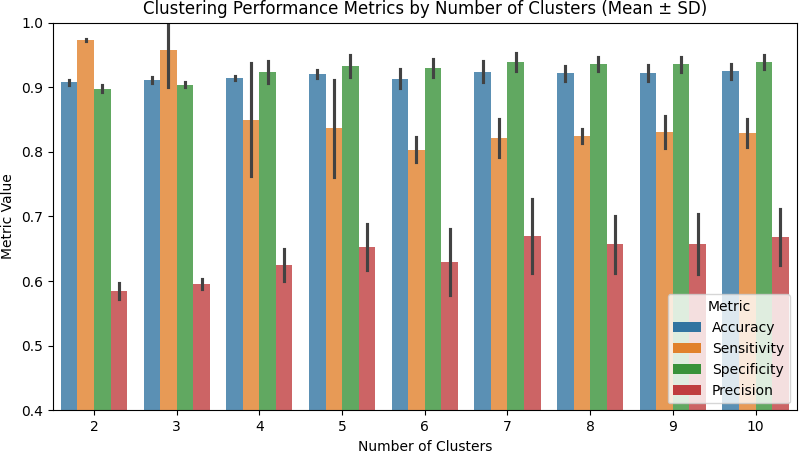


1. Performance with respect to Accuracy, Sensitivity, Specificity, and Precision of our QC method across 10 evaluation runs per each chosen number of clusters.


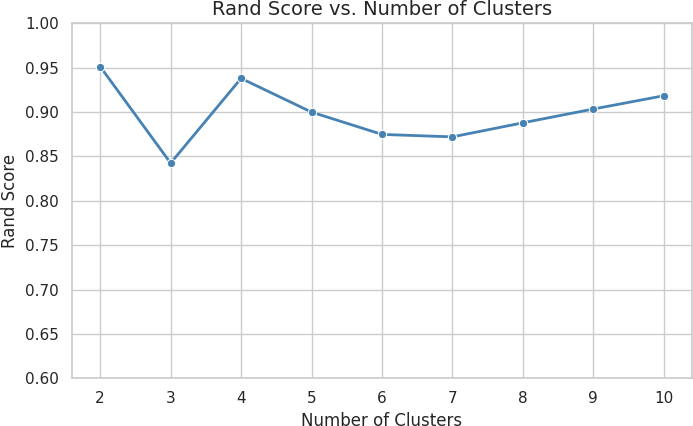


1. Rand Index scores measured across 1000 iterations of clustering for each chosen number of clusters. The reported numbers are the highest average Rand Index score that is acquired from comparing each clustering with the remaining 999 clusterings with the same cluster number.

Fig. 6: Figures indicating that the highest reliable sensitivity scores can be achieved by determining the number of clusters through Rand Index scoring.


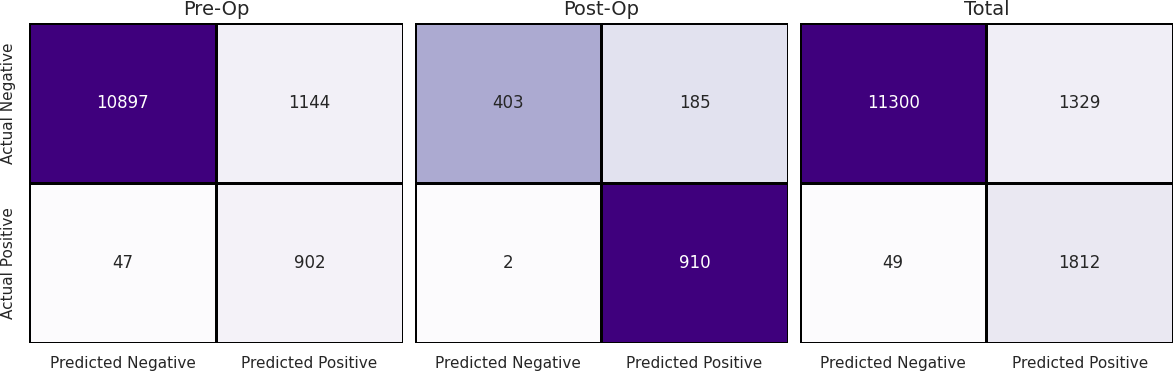


Fig. 7: Cumulative confusion matrices across our dataset consisting of pre- and post-operative masks. Our method correctly classifies the masks generated in- correctly from post-operative cases, while maintaining a low FN rate.

Table 5: The mathematical formulations in 3D-space of the various features used to quantify the properties of the brain mask along with their physical relevance.

| **Feature** | **Mathematical Representation** | **Relevance** |
| --- | --- | --- |
| Number of  Pixels | *Npix* | Highlights resolution differ- ences in volume. |
| Volume 54 | *V* = *Npix* ∗ *pixel.volume* | Gives good estimate of total  size. |
| Perimeter 55 | *P* (*X*) = *π* L *ck χ*(*X* ∩ *L_k_*)  *k λk* | Highlights resolution differ-  ences in surface area. |
| Surface Area | *A*(*X*) = *P* (*X*) ∗ *pixel.volume* | Provides a degree of surface  roughness and shape, aside from size estimate. |
| Equivalent  Spherical Radius | *V* ∗*Γ* ( 5 ) 1*/*3  *Req* = ^2^  *π*3*/*2 | Related to total size |
| Equivalent  Spherical Perimeter | *Peq* =  ^3^*^V^*  *Req* | Related to total size |
| Ellipsoid  Diameter | *Di* = 2*Req* j  *λi*  *ell* det(*σG*) | Provides a degree of shape,  size and pixel scattering. |
| Oriented  Bounding Box  Size 56 | *F^i^* = max*_p_*_¯_*_,q_*_¯_ (*p*¯ − *q*¯) · *e_λ,i_*,  *box*  where *p*¯*, q*¯ ∈ *Pixel.positions* | Provides a degree of shape, size and pixel scattering. |
| Pixels on  Border 54 | *Npix.border* =  \|{*p* : *p* ∈ Pixels*, onImageBorder(p)*}\| | Number of pixels that are  on the edge of an image. Can vary depending on size, shape, and scan protocol. |
| Perimeter  on Border | *Pborder* = *Npix.border* ∗ *pixel.volume* | Same as *N_pix.border_*, but  takes resolution into ac- count. |
| Perimeter  on Border Ratio | *Pratio* = *Pborder*  *P* | Potential insights into scan protocol and mask shape. |
| Feret Diameter 57 | *D_f_* = max*_p_*_¯_*_,q_*_¯_ \| [*p*¯ − *q*¯] *s*¯*^T^* \|, where *p*¯*, q*¯ ∈ *Pixel.positions*  and *s*¯ is the vector for pixel size | Captures a degree of size as  well as shape, by providing the euclidean distance of the two most distant pixels of a mask. |
| Elongation | *Felong* = j *λminor*  *λmajor* | Captures how much longer  the mask is than it is wide, by comparing its widest axis with its second widest axis. |
| Flatness | *Fflat* = j  *λleast*  *λmajor* | Captures how flat a mask is,  by comparing its widest axis with its smallest axis. |
| Roundness | *F* = *Rsph round P* | Measure of how of sphere-  like the mask is. |
| Sphericity | 2 1*/*3  *Fsph* = (36*πV* )  *A* | Measure of how of sphere-  like the mask is. |
| Spherical  Disproportion | *Fdis* = *A*  (36*πV* 2 )1*/*3 | Measure of how of sphere-  like the mask is. |
| Compactness 1 | *Fcomp.*1 = *V*  *π*1*/*2 *A*3*/*2 | Measure of how of sphere-  like the mask is. |
| Compactness 2 | 2  *Fcomp.*2 = 36*πV*  *A*3 | Measure of how of sphere-  like the mask is. |

Table 6: Performance of our methodology for BET, HD-BET, and BrainMaGe across T1, T2, T1Gd, and FLAIR modalities as well as their fusions on the Duke Dataset. Performance evaluation was separated by data-type and the sanity set spanned subjects from accross our entire dataset, identical to Table (Table 2 from paper). Paired two-tailed t-tests were performed to compare each performance metric between Pre-Op and Post-Op conditions across our 15 method-modality combinations.

| **Metric** | **Pre-Op** | **Post-Op** | **Total** | **t-statistic** | **p-value** |
| --- | --- | --- | --- | --- | --- |
| Accuracy | 0.8140 | 0.8753 | 0.8446 | -3.3134 | 0.0056 |
| Sensitivity | 0.9515 | 0.9978 | 0.9833 | -4.5665 | 0.0038 |
| Specificity | 0.7617 | 0.6853 | 0.7349 | 2.6202 | 0.0211 |
| Precision | 0.6027 | 0.8310 | 0.7458 | -3.8107 | 0.0021 |
